# Sleep and physical activity – the dynamics of bi-directional influences over a fortnight

**DOI:** 10.1186/s12889-022-13586-y

**Published:** 2022-06-10

**Authors:** Anu-Katriina Pesonen, Michal Kahn, Liisa Kuula, Topi Korhonen, Leena Leinonen, Kaisu Martinmäki, Michael Gradisar, Jari Lipsanen

**Affiliations:** 1 SleepWell Research Program, Faculty of Medicine, University of Helsinki, Helsinki, Finland; 2 Department of Psychology and Logopedics, Faculty of Medicine, University of Helsinki, Helsinki, Finland; 3 College of Education, Psychology and Social Work, Flinders University, Adelaide, SA Australia; 4 Polar Electro Oy, Polar Research Center, Kempele, Finland

**Keywords:** Physical activity, Sleep duration, Sleep quality, Big-data, Time-series, Cross-lagged

## Abstract

**Study objectives:**

The day-to-next day predictions between physical activity (PA) and sleep are not well known, although they are crucial for advancing public health by delivering valid sleep and physical activity recommendations. We used Big Data to examine cross-lagged time-series of sleep and PA over 14 days and nights.

**Methods:**

Bi-directional cross-lagged autoregressive pathways over 153,154 days and nights from 12,638 Polar watch users aged 18–60 years (M = 40.1 SD = 10.1; 44.5% female) were analyzed with cross-lagged panel data modeling (RI-CPL). We tested the effects of moderate-to-vigorous physical activity (MVPA) vs. high intensity PA (vigorous, VPA) on sleep duration and quality, and vice versa.

**Results:**

Within-subject results showed that more minutes spent in VPA the previous day was associated with shorter sleep duration the next night, whereas no effect was observed for MVPA. Longer sleep duration the previous night was associated with less MVPA but more VPA the next day. Neither MVPA nor VPA were associated with subsequent night’s sleep quality, but better quality of sleep predicted more MVPA and VPA the next day.

**Conclusions:**

Sleep duration and PA are bi-directionally linked, but only for vigorous physical activity. More time spent in VPA shortens sleep the next night, yet longer sleep duration increases VPA the next day. The results imply that a 24-h framing for the interrelations of sleep and physical activity is not sufficient – the dynamics can even extend beyond, and are activated specifically for the links between sleep duration and vigorous activity. The results challenge the view that sleep quality can be improved by increasing the amount of PA. Yet, better sleep quality can result in more PA the next day.

## Introduction

The question of the most functional balance between sleep and physical activity (PA) in an individual’s life is very relevant, as both sleep and PA contribute widely to health [1, 2]. The reciprocity of sleep and PA is suggested to include multiple physiological and psychological pathways [3]. For example, evidence suggests that the link between physical exercise and increased sleep duration could include interactions of circadian rhythm, metabolic, immune, thermoregulatory, vascular, mood and endocrine effects [3].

There exist numerous correlational studies linking different levels of daytime PA with sleep duration and quality during the subsequent night. The limitation in these studies is the focus on one direction of the association – usually the effect of PA on sleep but also the effect of sleep on PA [4–6]. The overall picture shows that acute or regular exercise typically has small-to-moderate beneficial effects on sleep duration and sleep quality and that better sleep quality may be associated with more PA the next day [4–6]. Yet, the results from individual studies vary greatly, due for example, methodological diversity. This includes variation in studied populations, measurement modes and time periods – also inter- and intra-individual analyses tend to give variation to the studied outcomes [4]. Furthermore, individuals have daily variation in both their sleep and PA, which indicates that measurement periods over multiple days are warranted.

Studies investigating the bi-directional effects, i.e., how sleep affects PA and vice versa in one within-subject design, are scarce. Of the studies using objective assessment of sleep and PA, one study reported that more daytime sedentary behaviour is associated with less total sleep time (TST) the subsequent night, and more TST in the preceding night is associated with less sedentary behaviour, but also less moderate-to-vigorous physical activity (MVPA) during the subsequent day [7]. These findings suggest that the intensity of PA may moderate the relationship between sleep duration and PA –longer sleep duration may decrease sedentary time but this does not necessarily result in more MVPA [7]. The other few studies using within-person analyses of the bi-directional effects between sleep and PA stem from youth samples. They show very mixed results, but provide evidence of the bi-directionality of the effects [8–10]. However, a recent systematic review and meta-analysis did not find strong support for bi-directionality, but it drew data from many unidirectional studies on both the effects of sleep on PA and PA on sleep, and was not based on modeling the bi-directionality within individuals [6].

Overall, the current understanding of the nature of the effects between sleep and PA is limited by lack of replicated outcomes, using small samples, methodological diversity in defining the variables of interest (general level of PA, sedentary behaviour, and level of the intensity of PA), emphasized focus on specific populations, as well as varying statistical approaches. There is also a lack of evidence on bi-directional effects between sleep and PA that are based on modeling their interdependency over a sufficient long time – we are not aware of studies extending up to 14 days, for example.

In this study, we take advantage of the data generated by the users of a commercially-available sport tracker allowing for exploration of *objective*sleep and PA measures in big-data, defined here as over 150 000 observed units (days + nights) [11, 12]. The main aim of the present study was then to explore the temporal dynamics between sleep duration, quality, and intensity of PA in a real-world big-data sample over a period of 14 days, taking into account the intensity of PA. More specifically, we aimed to test whether sleep-PA links vary as a function of PA intensity using within-subject modeling of objective metrics over this 2-week period.

## Methods

### Participants and procedures

Data from a total of 12,638 individuals aged 18–60 years (M = 40.1 SD = 10.1; 44.5% female) were included in this study. Data were obtained with Polar watches between January 2018 and December 2018, and included 153,154 measurement units (days + nights). The validity of the Polar watches in estimating MVPA has been tested against Actigraph, a device used widely for research purposes; MVPA time from wrist-worn Polar M430 correlated strongly with MVPA assessed with Actigraph placed at the waist (*r*= 0.75) [13]. Polar watch users agreed that their data can be used for Polar research and development purposes. Polar researchers handled the data anonymously and performed the statistical analysis for this study within the company. Randomly selected individuals were included in the sample if they had two consecutive weeks (14 days) of available sleep and physical activity data, as well as complete demographic data, including age, gender, height, weight, and country of residence. There were no missing values in the selected data set**.** Nights of sleep assessment were considered available if the duration of time from sleep onset to offset was between 4 and 13 h, and the total (true) sleep duration was between 3 and 13 h. Days of physical activity assessment were considered available if the device was worn for ≥ 10 h of wakefulness per 24-h day. Self-reported height and weight from the Polar user account settings were used to compute a BMI score for each participant (Range: 15–45; *M* = 25.7 *SD* = 4.1).

### Measures

Nighttime sleep and physical activity during the entire day were assessed using Polar devices (models A370, M430, M600, Vantage V, and Vantage M). These wrist-worn devices use validated proprietary algorithms to automatically translate biosignals into PA and sleep–wake metrics [14, 15]. For the purpose of the present study, derived sleep metrics included total sleep duration (calculated as the duration of time between sleep onset and offset, excluding wakefulness within that period), and sleep efficiency (calculated as the percent of total sleep duration out of the duration of time between sleep onset and offset). Sleep efficiency calculation thus does not include the sleep latency period and was used as a measure of sleep quality [16].

Physical activity levels were quantified per 30-s epochs and classified into one of the following categories: (1) moderate physical activity, with metabolic equivalent (MET) values between 2.95–5.95; (2) vigorous physical activity, with MET values between 5.95–8.75; or (3) near maximal physical activity, with MET values ≥ 8.75 [17]. In the present study, we used two PA variables, one including all activity from the moderate level upwards (MVPA) and one including activity from the vigorous level upwards (VPA).

### Statistical approach

**Fig. 1:**
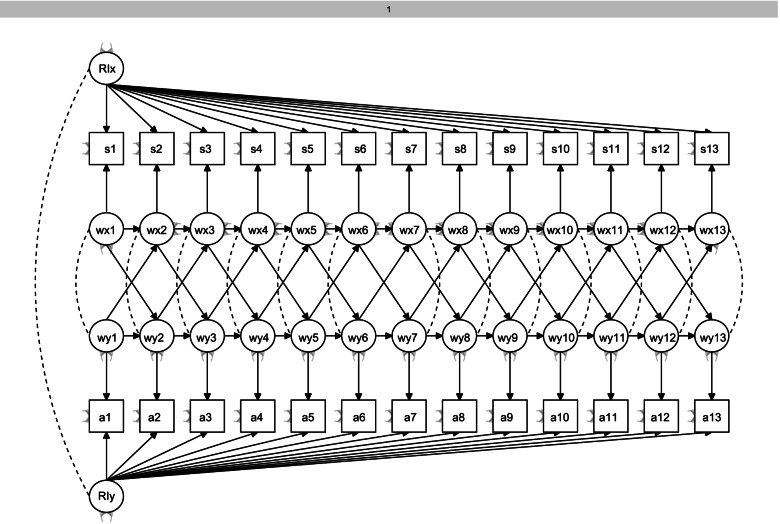
The cross-lagged panel model for sleep and PA. RIx and RIy = Between-subject latent intercept; s1…s14 = Observed sleep; a1….a14 = Observed PA; Wx = within-subject latent sleep excluding between-subject variation; Wy = within-subject latent PA excluding between-subject variation

We applied structural equation modeling to analyze the temporal associations between sleep and PA. We used random intercept cross-lagged panel data modeling (RI-CLPM) with the lavaan R-package [18]. This method allows for inclusion of the within-subject level in the model, unlike the traditional cross-lagged modeling [19]. Data were complete, with no missing values. To increase the stability of the models, cross-lagged paths were constrained to be equal in all models. Model fit was evaluated with the chi-square measure of exact fit, the Root Mean Square Error of Approximation (RMSEA) and its 95% confidence interval, the Comparative Fit Index (CFI), and Tucker-Lewis Index (TLI) [20]. In the RI-CLPM [19] each observed variable was regressed on its own latent factor, with each loading constrained at 1. This resulted in 14 within-person latent factors, reflecting the within-person variance within sleep and PA and in their cross-lagged associations. In addition, random intercept factors were added for sleep and PA variables to control for the between-person variance. This allowed taking into account the between-person differences for example in the habitual sleep features. The observed scores were the indicators of these random intercept factors, with all factor loadings constrained at 1. The BMI was added in the model as covariate. Figure 1 illustrates the cross-lagged panel model for sleep and PA.

## Results

### Characteristics of the focus variables

Table 1 shows means and standard deviations for the sleep and PA variables of interest in the sample. The correlation of MVPA and VPA sums was *r* = 0.45, *P* < 0.001. The correlation between sleep duration and efficiency was *r* = 0.27, *p* < 0.001.

**Table 1:** Descriptive statistics of the focus variables averaged by individual users over the 14 days’ measurement period

Variable	Mean (SD)
Moderate to vigorous activity (min)	144.5 (58.3)
Vigorous activity (min)	22.0 (20.1)
Sleep Duration (hours)	6.9 (0.7)
Sleep Efficiency (%)	93.4 (1.9)

### Results from the cross-lagged panel data models

Table 2 presents results from the two models (Model 1, MVPA; Model 2 VPA) involving sleep duration (i.e., sleep quantity). Model fits were good. Sleep duration, MVPA, and VPA showed significant within-subject temporal continuity in the auto-regressive associations. In Model 1, the cross-lagged within-subject associations showed that longer sleep duration was associated with less MVPA the next day, and there were no associations in the other direction. In Model 2, the associations between sleep and PA were bidirectional – higher VPA intensity predicted with shorter sleep duration the same night, and shorter sleep duration in turn predicted with less time spent in VPA. Adding BMI into the model did not alter the results (data not shown).

**Table 2:** Cross-lagged autoregressive models between sleep duration and PA

	Regression slopes for Model 1 (Actual Sleep Time and MVPA)				Regression slopes for Model 2 (Actual Sleep Time and VPA)			
	Estimate	Standard error	z	p	Estimate	Standard error	z	p
**Within-subject dependent variable: Actual Sleep Time**								
Autoregressive association: from sleep to sleep	0.01	0.00	3.89	< .0001	0.01	0.00	3.87	< .0001
Cross-lagged association: From PA to subsequent sleep	0.00	0.00	0.01	.990	-0.00	0.00	-3.65	< .0001
**Within-subject dependent variable: PA**								
Auto-regressive association: from PA to PA	0.06	0.00	14.34	< .0001	-0.06	0.00	-11.73	< .0001
Cross-lagged association: from sleep to subsequent PA	-1.92	0.17	-10.99	< .0001	0.23	0.07	3.14	.002
**Fit Indices**								
χ^2^	16,393.84				16,000.88			
CFI	0.87				0.81			
TLI	0.88				0.81			
RMSEA	0.05				0.05			
Scaled χ^2^ (df)	12,912.84 (418)			.000	10,656.40 (418)			.000

*PA* Physical Activity

*MVPA* Moderate to Vigorous Physical Activity

*VPA* Vigorous Physical Activity

*CFI* Comparative Fit Index

*TLI* Tucker-Lewin Index

*RMSEA* Root Mean Square Error of Approximation

Table 3 presents results from the two models (Model 1, MVPA; Model 2 VPA) involving sleep efficiency (i.e., sleep quality). Model fits were good. As in the previous models, sleep quality, MVPA, and VPA showed significant within-subject temporal continuity in the auto-regressive associations. There were no significant cross-lagged associations between PA and sleep quality in either model, indicating that the level of PA during the day did not affect the quality of sleep the next night. However, better quality of sleep was associated with both more MVPA and VPA during the subsequent day. When BMI was added into the model, the significant results did not change (data not shown).

**Table 3:** Cross-lagged autoregressive models between sleep quality and PA

	Regression slopes for Model 1 (Actual Sleep Percent and MVPA)				Regression slopes for Model 2 (Actual Sleep Percent and VPA)			
	Estimate	Standard error	z	p	Estimate	Standard error	z	p
**Within-subject dependent variable: Sleep Efficiency**								
Autoregressive association: from sleep to sleep	0.07	0.00	19.57	< .0001	0.07	0.00	19.56	< .0001
Cross-lagged association: From PA to subsequent sleep	0.00	0.00	1.32	.188	-0.00	0.00	-1.89	.058
**Within-subject dependent variable: PA**								
Auto-regressive association: from PA to PA	0.06	0.00	14.65	< .0001	-0.06	0.00	-11.73	< .0001
Cross-lagged association: from sleep to subsequent PA	0.98	0.09	10.60	< .0001	0.14	0.04	3.53	< .0001
**Fit Indices**								
χ^2^	10,469.02				10,096.44			
CFI	0.94				0.93			
TLI	0.95				0.93			
RMSEA	0.04				0.04			
Scaled χ^2^	7152.57 (394)			.000	5817.23 (394)			.000

*PA* Physical Activity

*MVPA* Moderate to Vigorous Physical Activity

*VPA* Vigorous Physical Activity

*CFI* Comparative Fit Index

*TLI* Tucker-Lewin Index

*RMSEA* Root Mean Square Error of Approximation

**Fig. 2:**
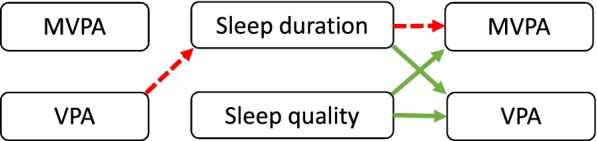
Narrative overview of the findings. Arrows refer to significant associations between physical activity and sleep. Red (dashed line) color indicates a negative association, and green (solid line) color a positive association

Figure 2 provides a narrative overview of the significant associations. We also converted the results to standardized coefficients to estimate the effect sizes. They were all small β < 0.1.

## Discussion

While the amount of studies on the relations of sleep and PA is large, only a handful have explored their mutual dynamics over a sufficiently long period of time, argued to be at least 12 days and nights at minimum [6]. We created a statistical framework to study the dynamic interconnections of sleep and PA over time. It included modeling the within-person bi-directionality of the associations providing a more accurate simulation of real-world effects than the more traditional uni-directional approaches. This understanding is central for public health knowledge as well as for intervention and planning of both exercise and sleep. We found stable within-subject patterns between sleep and PA over a 14-day period that were not similar in terms of sleep quantity and quality, and intensity of PA. While there is a consensus, that physical activity enhances sleep [4, 5] our study revealed a more fine-grained and dynamic account of this link – and showed that the opposite direction may be even more prevalent. The first contribution of this study was then to provide a clear statistical framework to study the flow of sleep and PA in big data that would allow further replications in other samples.

Our first finding pointed to a bi-directional effect between sleep and PA for sleep duration, but not for sleep quality. More time spent in VPA was associated with shorter nighttime sleep, whereas the amount of MVPA did not predict sleep duration in the subsequent night. In addition, longer sleep duration the previous night was associated with less MVPA but more VPA the next day. Thus, this bi-directional finding indicated that sleep duration is both a consequence of VPA and a predictor of VPA within oneself, and the effects are in opposing directions. This emphasises the role of sleep recovery in moderating the flow of intensive training from day to the next – however, a cross-lagged model with steps in 1 day/night at time does not respond to the question of average recovery time from VPA. Our study does not either respond to the question of how sleep architecture, such as the amount of slow wave sleep, is influenced by VPA during the preceding day. However, a prior experimental study showed no significant effect of a 1-h vigorous physical exercise with the basic sleep architecture during the following night [21]. However, the effect of PA can be concentrated on the first hours of sleep; VPA associated with a homeostatic recovery function during the first sleep cycle, with an increased delta wave power of SWS [21]. Thus, an intensive training episode during the day intensified the recovery function of SWS in the subsequent night. Whether this intensification could shorten the needed total sleep time, is not yet known. This is an intriguing hypothesis though.

While previous within-subject studies in adults are few, the link between longer sleep duration and a lesser amount of MVPA during the next day has been found also in a previous study [7]. A link between increased PA and shorter sleep the following night, a similar finding to ours, has been previously reported in school-aged children [8, 10] and in adults [6]. However, comparing the results is challenging, as the studied variables, intensity of PA, and study design varied. In addition, timing of the physical activity may matter—we have previously reported from this same data set that MVPA performed in the 3hbefore bedtime was associated with slightly longer sleep duration [22].

Our second finding demonstrates a unidirectional association between sleep quality and PA – such that a more continuous and better quality of sleep predicted more MVPA and VPA the subsequent day. In contrast, sleep quality was not affected by PA levels in the preceding day. This finding echoes our previous report from this same dataset, showing that MVPA performed during the evening hours before bedtime was unrelated with sleep quality [22]. However, there have been very few studies that have found better sleep quality (i.e., sleep efficiency) to precede such high levels of PA [6].

### Strengths and limitations

The strength of this study is in objective assessment of both PA and sleep, allowing a more reliable estimate of intensity of PA and quality of sleep, than gained by self-reports. A continuous measurement period of 14 days in a large population of 12,638 participants is another asset of the study, allowing detection of the dynamic effects between sleep and PA. Using a real-life naturalistic setting, where consumer wearables are regularly used by individuals for tracking their own sleep and activity, which increases the ecological validity of the study. The statistical approach is also a strength – implementing a validated analytic strategy may help in consolidating research approaches in this area. Notwithstanding these strengths, our model is susceptible to an influence from a multitude of individual or environmental factors such as sleep disorders, medical conditions, and participants’ training programs that were not available in the data. We did not analyze potential napping, which may confound the results. Also, we did not specifically look at low intensity activity, which may also have a beneficial impact on sleep [23]. We chose to investigate lag-1 models between sleep and physical activity, as evidence on these immediate bi-directional effects is currently lacking. We are aware that longer lags, such as lag-7, between sleep and PA have been suggested, based on the found cross-lagged associations between sleep and a wide range of waking health behaviours, such as drinking, smoking and caffeine consumption [24]. However, exercising did not influence sleep in this lag-7 study [24]. Finally, the generalisability of our findings may be limited by selection bias, as users of wearable fitness tracker devices may more likely engage in health promoting behaviours compared to the general population. This study did not either examine the effects of age and sex as no hypotheses could be formulated based on prior reviews [3, 6].

## Conclusions

We showed that sleep duration, but not sleep quality, is in bi-directional relation with PA, although the effects sizes were small. Above the observed associations, our study proposes a feasible conceptual and statistical framework for studying the interrelations of sleep and PA. We suggest that the future studies on the topic should apply time-series modeling that is statistically and conceptually above of testing only the unidirectional and/or between-person links. From the public health perspective, the results challenge public health recommendations of using physical activity as a means to improve sleep. Also, this approach broadens the framework of using a schematic 24- hours activity and sleep window to consider health behaviors in wider time perspectives. This would be especially important for developing exercise planning at a more vigorous activity level, where recovery time is essential. Paradoxically, while the findings rely on a big data sample, a personalized context may provide their greatest exploitation opportunities, for example through wearable algorithms assisting individuals to find their best sleep and PA rhythms. Towards this aim, future studies could also benefit from teaming up of wearable industry and academic research. 

## Data Availability

Polar provided the data and performed the statistical analysis for this study. The data that support the findings of this study are not publicly available because the dataset consisted of unique data from Polar customers. Providing the full set of data would compromise privacy and data security of the customer data. All data analysis scripts are available at request from jari.lipsanen@helsinki.fi.
